# Multi‐faceted role of pyroptosis mediated by inflammasome in liver fibrosis

**DOI:** 10.1111/jcmm.17277

**Published:** 2022-04-12

**Authors:** Hui Yang, Juan Wang, Zhen‐Guo Liu

**Affiliations:** ^1^ Department of Infectious Disease The Third Xiangya hospital Central South University Changsha China; ^2^ Hunan Key Laboratory of Viral Hepatitis Xiyang Hospital Central South University Changsha China

**Keywords:** inflammasomes, liver fibrosis, pyroptosis

## Abstract

Liver fibrosis is a reversible pathological overreaction during the self‐repair of liver injuries, and it is the common period of chronic liver diseases induced by different pathogenesis progress into cirrhosis and even hepatocellular carcinoma. Pyroptosis, a novel form of programmed cell death, is reported to take part in the pathogenesis and progression of acute or chronic liver diseases and liver fibrosis. Caspase‐1 dependent canonical pathway and caspase‐4/‐5/‐11 mediated noncanonical pathway are the two signalling pathways to induce pyroptosis. The activation of inflammasomes under the stimulation of pathogenic microorganisms and danger signals can initiate the pyroptotic pathway and release large amounts of proinflammatory and profibrotic cytokines. This article comprehensively summarizes recent researches focused on the mechanism of pyroptosis and its role in major hepatic cells, which can provide potential therapeutic strategies for liver fibrosis.

## 1 INTRODUCTION

Liver fibrosis is a common progressive pathological process of chronic liver injury caused by multifarious aetiologies. The imbalance between the generation and degradation of extracellular matrix (ECM) in the liver leads to excessive deposition of ECM. Activated hepatic stellate cells (HSCs) are the chief source of ECM and the central link of liver fibrosis. In addition, inflammation, as one of the initiating factors of liver fibrosis, plays a leading role in fibrosis through the interaction of inflammatory cells, cytokines, and related signalling pathways.^1^ Fibrogenesis could promote wound healing in the early stage of injury, but the ongoing fibrosis can result in physiological architectural changes of the liver and finally transition to cirrhosis or hepatocellular carcinoma. Previous studies considered liver fibrosis as an irreversible process, but more and more researches have confirmed that fibrotic liver architecture can be improved gradually in human and mouse models when injured stimulus is removed or suppressed.^2^

Pyroptosis is a newly discovered inflammatory programmed cell death mediated by inflammasome which differs from other types of cell death, such as apoptosis, necrosis and autophagy. The remarkable features of pyroptosis include the formation of pore on plasma membranes, cell swelling and rupture and subsequent release of inflammatory cytokines and intracellular materials.^3^ Pyroptosis is closely related to defence against microbial infections, and is also involved in many other clinic diseases, such as cancers, cardiovascular diseases and autoimmune diseases.^4^ Besides, it has been proved that pyroptosis plays critical role in various liver diseases and the development of liver fibrosis.^5^, ^6^ Studies have shown that inhibiting pyroptosis of hepatic cells can alleviate cell damage and inflammatory reaction. Meanwhile, inflammatory cytokines secreted by pyroptotic cells can induce the activation of HSCs and ultimately promote the occurrence and development of liver fibrosis.^7^, ^8^ This review summarizes the mechanisms of pyroptosis and its role in liver fibrosis, and it may provide a theoretical basis for the anti‐fibrosis treatment.

## 2 PYROPTOSIS

Pyroptosis is considered as an innate immune response triggered by pathogenic microorganisms such as bacteria, virus, fungus and endogenous cellular contents or cytokines. As a kind of proinflammatory cell death, moderate pyroptosis can improve the self‐protection ability of cells and contribute to the timely elimination of pathogenic microorganisms and infected cells. However, numerous evidence revealed that excessive pyroptosis could induce sterile inflammation, result in superabundant cell death, serious tissue damage and organ dysfunction, and eventually to some certain diseases.^9^

Pyroptosis was discovered in macrophages after Shigella infection initially, which was recognized as apoptosis mistakenly due to they had similar morphological characteristics to some degree.^10^ Then, the alike phenomenon was also seen in Salmonella‐infected macrophages in 1999.^11^ In 2000, the term ‘Pyroptosis’ was originally proposed, which was defined as caspase‐1 dependent inflammatory programmed cell death.^12^ Because many studies suggested that caspase‐1 inhibitors or gene knockout could interrupt this type of cell death, while caspase‐3 had no similar effect.^13^ As gasdermin‐D (GSDMD) was gradually discovered as an important executor of pyroptosis, the definition of pyroptosis was revised as gasdermin‐mediated programmed necrosis.^14^

Gasdermin‐D, belonging to the gasdermin family, is the executor of pyroptosis. Other members of this family including gasdermin A, B, C and E, also participate in the process of pyroptosis.^15^ Almost all gasdermins contain amino‐terminal pore‐forming (N) domain and carboxy‐terminal (C) domain. Recent studies showed gasdermin N domain could induce cell pyroptosis and eliminate pathogenic bacteria.^16^ The caspase‐1/11 in mice and caspase‐1/4/5 in human are activated following pathogen recognition and inflammasome formation, subsequently cleaving GSDMD and leading to the formation of pore on cell membrane and the release of cytokines. Inflammatory mediators induce inflammatory responses by recruiting immune cells. In addition, pores can increase the permeability of cell membranes. Electrolytes inside and outside the cell membranes are imbalanced, resulting in electrochemical and osmotic gradients. The cell membrane loses the ability to regulate substances in and out, and the entry of water leads to the expansion and rupture of the cell membrane.^17^ There are two pathways to induce pyroptosis including caspase‐1 dependent canonical pathway and caspase 4/5/11 dependent noncanonical pathway.^18^

### 2.1 Canonical pathway

The canonical pyroptosis is triggered by host pattern recognition receptors (PRRs) after recognizing damage‐associated molecular patterns (DAMPs) and pathogen‐associated molecular patterns (PAMPs).^19^ It is mediated by caspase‐1, which is the only discovered caspase participating in this pathway and is activated by inflammasomes. Inflammasomes are multiple complexes that comprise Nod‐like receptor (NLR), apoptosis‐associated speck‐like protein containing CARD (ASC) and pro‐caspase‐1.^20^ ASC, as the coupling element of inflammasome complexes, consists of a caspase recruitment domain (CARD) interacting with pro‐casepase‐1 and a pyrin domain (PYD) combined with PRRs. The activation of inflammasomes can recruit pro‐caspase‐1 and cleave it into active caspase‐1, then caspase‐1 further lysis Pro‐IL‐1β and pro‐IL‐18 into mature IL‐1β and IL‐18. Besides, activated caspase‐1 can also cleave GSDMD into biologically active GSDMD N‐terminal fragment, which can form pores on cytomembrane, then secrete intracellular contents including IL‐1β and IL‐18 and finally induce pyroptosis.^5^, ^21^

Until now, the main five kinds of inflammasomes researched widely in pyroptosis are absent in melanoma 2 (AIM2), NLRP1, NLRP3 and NLRC4. Among those inflammasomes, NLRP3 is the most important trigger of pyroptosis.^22^ NLRP3 could be activated by a serial of DAMPs and PAMPs, such as bacteria, viruses and fungus. The moderate activation of NLRP3 inflammasome presents protective effects, while uncontrollable activation will result in chronic inflammation and other pathological changes. The AIM2 inflammasome, a cytosolic receptor, senses self and microbial double‐stranded DNA through the carboxy‐terminal HIN200 domain.^23^ The only well‐known trigger of NLRP1 activation is anthrax lethal toxin excreted by anthracis, and NLRP1 activation can defence against anthracis infection via inducing pyroptosis.^15^ The NLRC4 inflammasome in macrophages and dendritic cells recognizes cytosolic bacterial flagellin to exert antimicrobial effects.^4^ Subsequently, inflammasomes combine with ASC, then cleave pro‐caspase‐1 into active caspase‐1. Interestingly, the NLRC4 can directly recruit pro‐caspase‐1 without ASC.^5^ In addition, NLRP6 recognizes bacteria and microbial metabolites, and NLRP9 binds to short dsRNA via RNA helicase DEAH‐box helicase 9. Pyrin, another form of the inflammasome, responds to pathogen‐induced modification of Rho GTPases.^24^

### 2.2 Noncanonical pathway

The activation of noncanonical inflammasome pathway dependent on caspase 4/5/11 other than caspase‐1. Caspase‐11 mainly exists in mice, while caspase‐4/5 exists in human.^25^ The CARD domain of caspase‐4/5/11 combines with lipopolysaccharide (LPS) and promote its own oligomerization and activation. The activated caspase‐4/5/11 cleaves GADMD into biologically active GSDMD‐NT, which can form pores on cytomembrane and induce pyroptosis directly.^26^ GSDMD‐NT can also cause the activation of caspase‐1‐dependent NLRP3 inflammasome, leading to the secretion of IL‐1β and IL‐18, which is related to promote the canonical pyroptotic pathway indirectly.^27^

**FIGURE 1:**
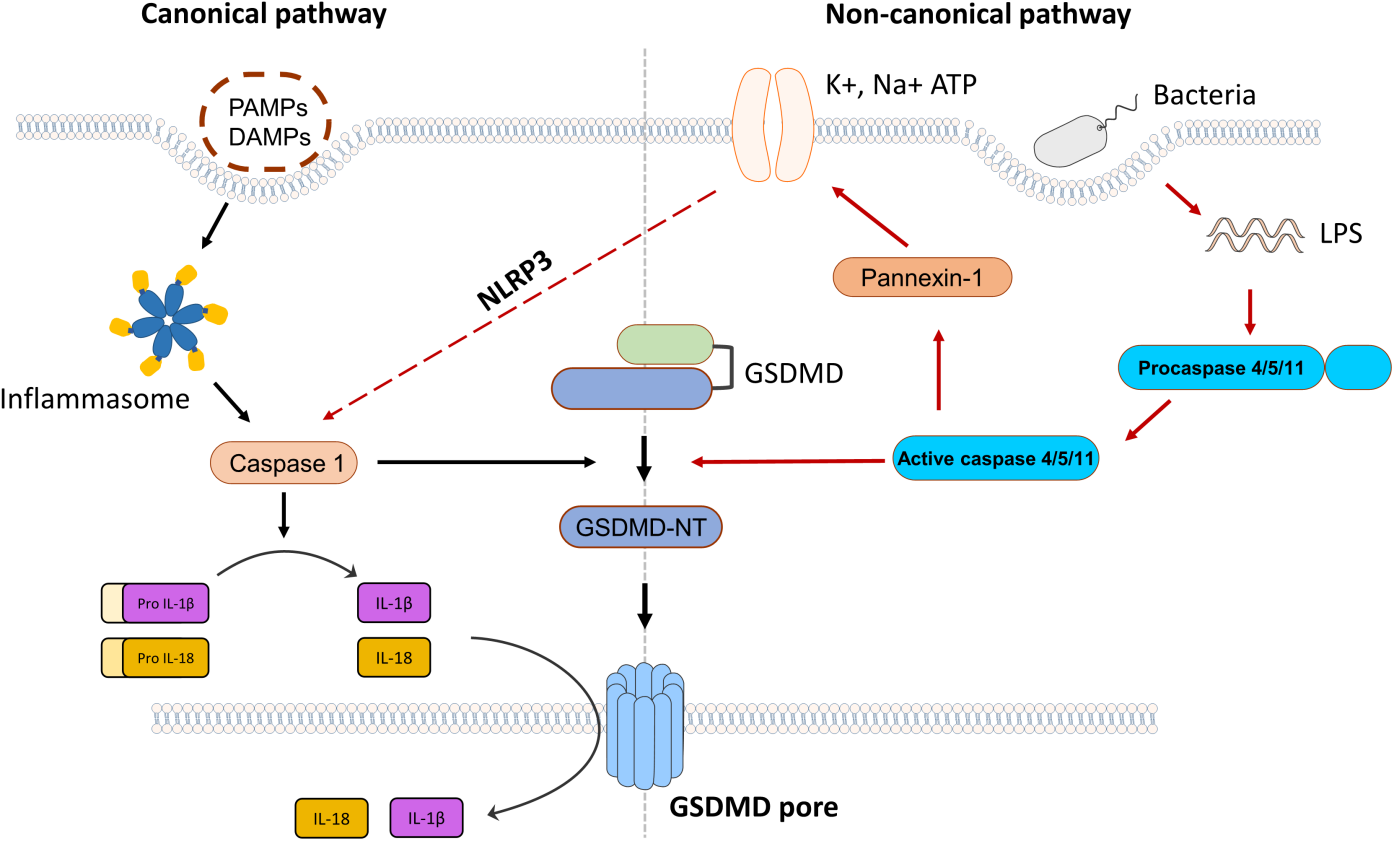
Canonical and noncanonical pathways of pyroptosis: The canonical pyroptosis is mediated by caspase‐1 after recognizing PAMPs or DAMPs, while the initiation of noncanonical pyroptosis is dependent on caspase 4/5/11. GADMD can be cleaved by these caspases into biologically active GSDMD‐NT, which can form pores on cytomembrane and induce pyroptosis. Caspase‐1 also lysis Pro‐IL‐1β and pro‐IL‐18 into mature IL‐1β and IL‐18, and caspase‐4/5/11 can activate pannexin‐1 and P2X7. Besides, the two pyroptoticpathways can interact each other via NLRP3 inflammasome

Some novel mechanisms have been discovered with further researches, and one of the most remarkable is Pannexin‐1. Pannexin‐1 is a channel protein of cellular membrane that controls small molecules in and out. Caspase‐4/5/11 can trigger pannexin‐1 and release ATP into extracellular space, then activating P2X purine receptor 7 (P2X7), which finally prompting the formation of small pores in cytomembrane and occurrence of pyroptosis.^28^ In addition, the opening of P2X7 will facilitate intracellular ion efflux including K^+^, Na^+^ and Ca^2+^.^29^ The balance of ion inside and outside the cell is disrupted after ion efflux, and then, the cell undergoes osmotic swelling and rupture, eventually leading to cell death. Moreover, ion efflux can induce NLRP3/caspase‐1 activation and pyroptosis. Therefore, the noncanonical and canonical pathways are linked together via GSDMD and pannexin‐1(Figure 1).

## 3 MAIN HEPATIC CELLS UNDERGOING PYROPTOSIS IN LIVER FIBROSIS

Many aetiologies can lead to liver fibrosis, such as hepatotropic virus infection, alcoholic steatohepatitis (ASH), non‐alcoholic steatohepatitis (NASH), autoimmune liver disease and hereditary disease. The role of pyroptosis has been investigated in above chronic liver diseases. The studies suggest that inflammatory reaction accompanies the whole process of liver fibrosis. Inflammasomes, the important regulators of fibrosis, can induce the activation of caspase‐1, and the release of IL‐1β and IL‐18, as well as trigger pyroptotic cell death under the injury stimulations.^20^ These processes can initiate a wound healing response, but can also aggravate tissue damage by activating HSCs and lead to abnormal scar formation.

**FIGURE 2:**
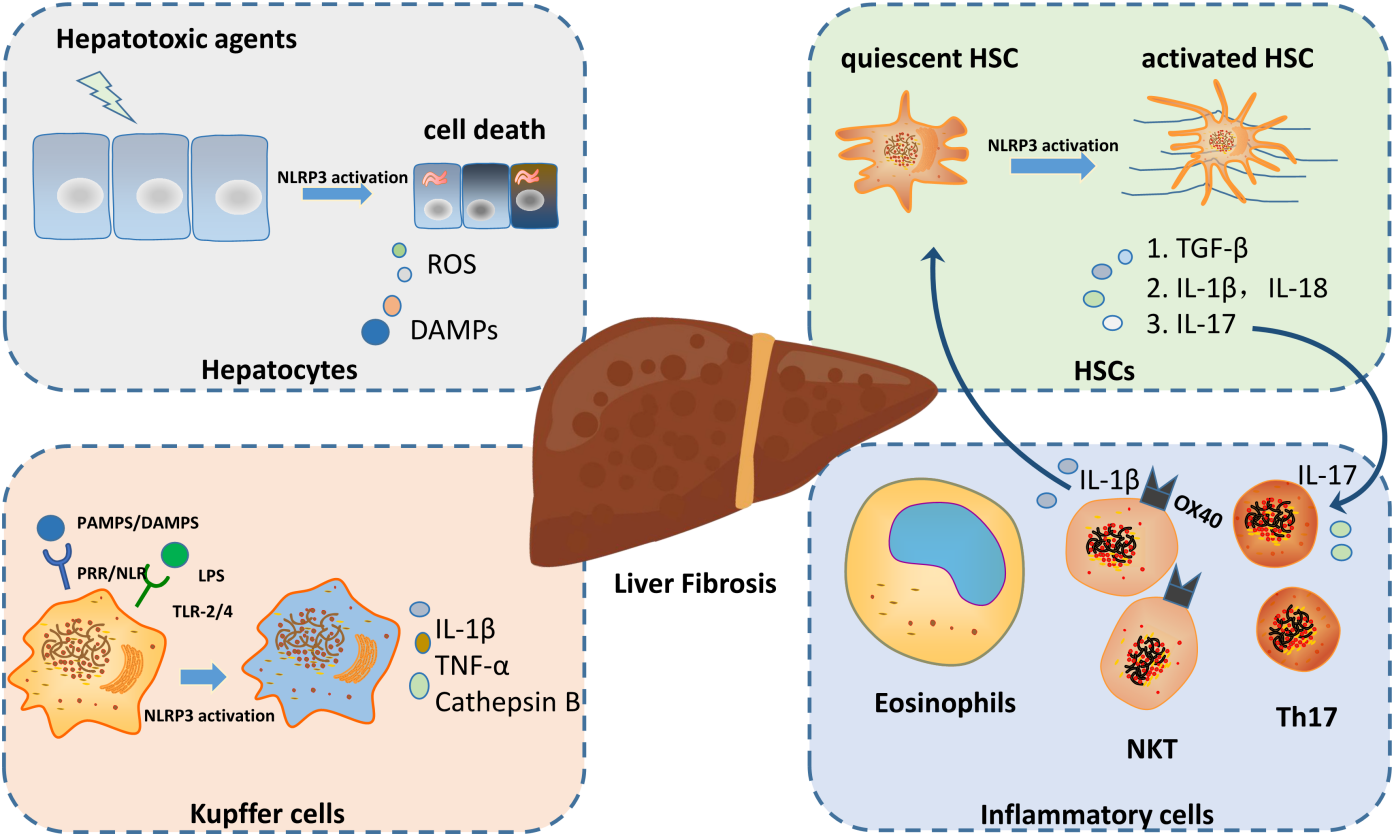
Multi‐faceted role of pyroptosis in liver fibrosis: Liver fibrogenesis is a dynamic process that requires the interaction of numerous cells, cytokines and signaling pathways. Hepatic cells are composed of hepatocytes and nonparenchymal cells such as HSCs, Kupffer cells and other immune cells. The occurrence of pyroptosis can exhibit in these cells through various molecular mechanisms, which affect the expression of inflammatory and fibrotic cytokines and ultimately regulate liver fibrosis

Liver fibrogenesis is a dynamic process that requires the interaction of various cells, cytokines and signalling pathways. Hepatic cells are composed of hepatocytes and nonparenchymal cells such as HSCs, Kupffer cells and other immune cells. These different cells elements are involved in many important physiological functions of the liver. The pyroptosis exerts pro‐fibrotic effect via regulating cell element, as inflammasomes can be found increased in macrophages obviously, expressed in HSCs and hepatocytes moderately.^30^ (Figure 2).

### 3.1 Hepatic stellate cells (HSCs)

The activation and transdifferentiation of HSCs into myofibroblasts (MFBs) are the most predominant step of hepatic fibrosis. HSCs are considered as the major source of ECM.^31^ HSCs are quiescent or inactivated in the normal liver and account for 15% of total resident cells, which localize in the subendothelial space of Disse between hepatocytes and endothelial cells, and the quiescent HSCs is rich in lipid droplets.^32^ Under the continuous pathogenic stimuli, HSCs are activated and increasingly lose the lipid droplets, and then transdifferentiate into proliferative and contractile MFBs. The activation of HSCs involves two steps—initiation stage and perpetuation stage. In the first stage, HSCs undergo the regulation of gene expression and phenotype changes after the stimulation of products released by damaged hepatocytes, Kupffer cells and other hepatic cells. Chronic injury and persistent stimulation result in the perpetuation period.^33^ Significantly, if the pathogenic stimulus are removed timely, most activated HSCs could return into the inactive phenotype or undergo senescence and apoptosis, which finally lead to liver fibrosis regression.^34^

NLRP3 inflammasome also exists in HSCs and can be activated, therefore, assisting in activating HSCs and exacerbating development of fibrosis.^35^ Monosodium urate crystals, as the well‐known NLRP3 inflammasome activator, can upregulate the expression of TGF‐β and collagen I in both human HSC lines and primary mouse HSCs.^36^ Another study revealed that the continuous activation of NLRP3 in HSC can result in significant increase of α‐SMA positive cells, larger sirius red staining area, and exhibit severe liver fibrosis by using a model with selective expression of mutant hyperactive NLRP3 in HSC.^37^ Similarly, NLRP3 activation in HSCs also leads to phenotype switch into MFBs induce higher expression of fibrotic biomarker, such as α‐SMA, TGF‐β and ECM, finally aggravating the pathological process of liver inflammation and fibrosis.^38^ Matured IL‐1β and IL‐18 are released after the activation of NLRP3. These two cytokines have been demonstrated to participate in inflammatory and fibrotic process in multiple chronic liver disease. Under long‐term inflammatory stimulation, IL‐1β can recruit immune cells such as monocytes, macrophages and dendritic cells into the liver, which interact with HSCs to promote the progression of liver fibrosis.^39^ In contrast, Li et al.^40^ discovered that a large amount of HSCs pyroptosis can alleviate fibrosis. ASIC1a, a subunit of acid‐sensitive ion channels, highly expressed in acid‐induced active HSCs, can inhibit pyroptosis of HSCs. They proved that suppressing or silencing ASIC1a could relieve liver fibrosis via promoting HSCs pyroptosis. Until now, there are only a few studies to investigate the exact effects of HSC pyroptosis on liver fibrosis; so further exploration is needed to uncover the intrinsic connection.

Besides, the intracellular interaction in liver can activate HSCs. The cytokines released by hepatocytes and Kupffer cells participate in HSCs activation and liver fibrosis formation. After the persistent injury, numerous hepatic cells underwent multiple ways of cell death including pyroptosis, which followed by the release of intracellular substances. These molecules act as endogenous danger signals to stimulate and activate inflammasomes in the neighbouring cells, such as innate immune cells and HSCs, which further promote the secretion of cytokines and other danger signals. Moreover, this paracrine signalling between these cells can induce HSCs activation, collagen deposition and liver fibrogenesis.^41^ Moreover, the activation of NLRP3/caspase‐1 in HSCs can induce Th17 cell differentiation and increase the secretion of IL‐17.^42^ In the liver tissue from hepatitis B virus related cirrhosis patient, the expression of IL‐17 was significantly upregulated.^43^ By using Nlrp3^A350V^ knock‐in mice breeding onto *IL17a* knockout backgrounds, Wree discovered that NLRP3 overexpression mice presented severe liver inflammatory and fibrotic changes, while *IL17a* lacking mutants exhibited improvement of fibrosis.^44^ Moreover, in cholestatic and hepatotoxic mice models of liver fibrosis, IL‐17 and its receptor IL‐17RA were also highly expressed. It further discovered that IL‐17 can induce Kupffer cells to express pro‐inflammatory and fibrogenic factors such as IL‐6, TNF‐α and TGF‐β1. On the contrary, IL‐17 also directly stimulates collagen I production in HSCs via activation of the STAT3 signalling pathway, ultimately promoting liver fibrosis.^45^

### 3.2 Macrophages

Hepatic macrophages comprise the inherent Kupffer cells and bone marrow derived macrophages,^31^ are the main regulatory cells in the process of liver fibrosis. Macrophages activate resting HSCs to promote fibrogenesis, and can also induce apoptosis of activated HSCs and degradation of collagen to reverse liver fibrosis. Macrophages present different phenotypes depending on the microenvironment and also play diverse regulatory roles in inflammation and fibrosis. The pro‐fibrotic macrophages (M1) release proinflammatory cytokines, such as IL‐6, TNF‐α and IL‐1β under the stimulation of interferon‐γ or LPS to aggravate liver injury and induce fibrosis, while the immunosuppressive macrophages (M2) generate anti‐inflammatory factors including IL‐10, PDGF and TGF‐β under the stimulation of IL‐4, IL‐10 and IL‐13.^46^ As the injury worsens, the advantaged macrophages gradually switch to the M2 phenotype and exhibit anti‐inflammatory effect.

Kupffer cells, accounting for 30% of sinusoidal cells, are liver resident mesenchymal macrophages and are crucial for liver inflammation and fibrosis. They can be activated by variety stimulus through endocytosis. On the one hand, Kupffer cells participate prominently in engulfing foreign pathogens, eliminating endotoxins, presenting antigens and regulating immune response, thus protecting hepatocytes. On the other hand, Kupffer cells also play the proinflammatory and profibrotic role in the early stage of injury. The activated KCs secrete inflammatory cytokines including TNFα, IL‐1β and chemokine, which activate HSCs and recruit circulating macrophages. KCs also promote inflammatory response via upregulating nuclear factor kappa‐B (NF‐κB) in HSCs.^47^

Kupffer cells can recognize PAMPs and DAMPs through PRR and NLR, and further promote the production of inflammatory cytokines.^48^ Cytokines secreted by Kupffer cells can mediate inflammatory cascade responses, and eventually inducing tissue remodelling and fibrosis. Besides, Kupffer cells can produce large amounts of ROS, which can promote HSCs activation, collagen synthesis and lead to liver fibrosis. NLRP3 inflammasome is highly expressed in Kupffer cells in liver. Kupffer cells are considered as the primary source of IL‐1β and the main cell type of NLRP3 inflammasome activation, and caspase‐1‐knockout Kupffer cells cannot secret IL‐1β under LPS stimulation.^49^ This suggest that Kupffer cells play a vital role in the activation of inflammasome. A research has shown that the selective blockage of Kupffer cells with gadolinium chloride, a macrophage inhibitor, could significantly reduce the expression of NLRP3 inflammasome and the activation of related signalling pathways, and further inhibit caspase‐1 activation and the progress of liver fibrosis.^50^ Vahid M found that rare‐earth oxide nanoparticles can induce pyroptosis via mediating lysosomal damage and activating NLRP3/caspase‐1 in primary Kupffer cells, bone marrow‐derived macrophages and other macrophages lines, while the features of pyroptosis was not observed in hepatocytes.^51^

Lipopolysaccharide can activate macrophage via Toll‐like receptor 4(TLR4)‐independent pathway and activating caspase‐11, then activated caspase‐11 could cleave GSDMD to induce both noncanonical pyroptosis and activate NLRP3‐dependent caspase‐1, and finally secreting numerous cytokines especially IL‐1β and IL‐18.^26^, ^48^, ^52^ As Kupffer cells constitute almost 80% of tissue macrophages, the similar phenomenon may also be occurred in Kupffer cells. Not surprisingly, Feng et al.^53^ has also demonstrated that TLR4, the main ligand of LPS, drive Kupffer cells to produce various cytokines under LPS stimulation, thereby mediating liver fibrosis. LPS also binds to Toll‐like receptor 2 on Kupffer cells to promote NLRP3 activation, further activate caspase‐1 and induce pyroptosis of liver parenchymal cells. The followed amplified inflammatory response can stimulate the activation of HSC and secrete ECM, leading to the occurrence of liver fibrosis. Macrophages pyroptosis also participates in liver fibrogenesis in NAFLD and CCL4‐induced models.^54^, ^55^ In addition, the release of mitochondrial DNA can induce NLRP3 inflammasome activation in Kupffer cells and drive IL‐1β secretion in NASH mouse model.^56^ Furthermore, recent study reveals that LPS can lead to the expression of cathepsin B, which gradually activates caspase‐11 in Kupffer cells and induce noncanonical pyroptosis, resulting in liver damage.^57^

### 3.3 Hepatocytes

Hepatocytes, the parenchymal cells of the liver, occupying most of the resident cells of the liver and maintaining the liver structure, are the main targets of hepatitis viruses, alcohol, bile acid and other toxic substances.^42^ Under the attack of those hepatotoxic agents, hepatocytes produce a series of inflammatory mediators, lipids, hedgehog ligands, reactive oxygen species (ROS) and DAMPs following different cell death modes including necrosis, apoptosis, pyroptosis and autophagy. Then, it can promote HSCs activation directly or indirectly, and ultimately results in liver inflammation and fibrosis.^58^ The death of hepatocytes is a key element in chronic and acute liver disease.

Inflammasomes, especially NLRP3 exist moderately in hepatocytes, and the excessive activation and overexpression of NLRP3 in hepatocytes will trigger cell pyroptosis, leading to serious liver injury and fibrogenesis.^47^ Wree et al.^59^ found that hepatocytes pyroptosis induced by NLRP3 inflammasome is a critical element of liver fibrosis for the first time. They used global and myeloid‐specific mutant NLRP3 knock‐in mice models to assess the degree of pyroptosis and liver fibrosis. The results showed that hepatocytes separated from myeloid‐specific NLRP3 mice exhibit fewer pyroptosis, and the activation of myeloid‐specific NLRP3 can lead to less inflammation and fibrosis in liver compared with global mutant mice. Another study of this team further proved that hepatocytes pyroptosis and the release of inflammasome complex following NLRP3 overaction, and the extracellular inflammasome can be endocytosed by HSCs, finally resulting HSCs activation and promoting liver inflammation fibrosis in vivo and in vitro.^60^ In addition, Lebeaupin confirmed that the stimulation of LPS could induce endoplasmic reticulum stress, which further activates NLRP3 inflammasome, eventually resulting in hepatocytes pyroptosis in NAFLD mice model.^61^ In ASH animal models and patients, Heo also found alcohol can activate NLRP3 inflammasome and mediate pyropotosis in hepatocytes via overexpression of TXNIP.^62^

Hepatocyte pyropotosis induced by endogenous inflammasome activation can also aggravate inflammatory response and promote liver fibrosis. So, hepatocyte pyroptotic cell death may be a novel mechanism of NLRP3‐mediated liver injury and blocking the NLRP3 pathway could be a promising target to attenuate liver fibrosis.

### 3.4 Other immune cells

A large number of inflammatory cells and immune cells, especially macrophages and neutrophils, can be recruited to produce a series of cytokines after liver injury, thereby inducing inflammatory response even liver fibrosis. In addition, eosinophils can also be accumulated into the damaged area and are associated with some liver diseases.^63^, ^64^ As one of the important components of leukocytes, eosinophils not only have the function on killing pathogenic bacteria but also participate in immune defence response. These cells can release intracellular contents, cause tissue damage and promote inflammation. Besides, eosinophils are also reported to undergo pyroptosis to induce liver fibrosis. A study demonstrates that injured hepatocytes could recruit eosinophil and facilitate the secretion of IL‐1β and IL‐18 which can further induce hepatocytes death and HSCs activation in vivo and in mouse infected with Schistosome mansoni. It is also suggested that pyroptosis of infiltrating eosinophils participate in liver fibrosis owing to the above process can be restrained by caspase‐1 inhibitors.^65^ But the detail mechanisms that eosinophil pyroptosis regulates hepatic fibrogenesis needs to be further explored.

Natural killer T (NKT) cells, as a kind of immune cells, reside abundantly in liver. It was reported that the accumulated NKT cells induce HSCs transdifferentiation into MFBs and promote fibrogenesis via Hedgehog pathway in NASH models established with methionine choline deficient diets.^66^ A recent study reveals that hepatic invariant NKT cells, a subset of NKT cells, can exert pyroptotic cell death. Lan et al.^67^ found that the activation of caspase‐1 in NKT cells also leads to the production of IL‐1β and GSDMD, subsequently forming a membrane pore and inducing pyroptosis, and this process was mediated by a costimulatory TNF superfamily receptor called OX40 stimulation and subsequent recruitment of MALT1. It suggests that pyroptosis of NKT cells is triggered by OX40 other than activation of inflammasomes compared with pyroptois of macrophages, though both of them can aggravating liver inflammation and injury. These results provide new evidence for the mechanism and prospective therapeutic target of liver fibrosis.

Liver is composed of a variety of cells including hepatocytes and nonparenchymal cells. In addition to the cells described in this review, inflammasome is also expressed in liver sinusoidal endothelial cells, myofibroblasts, dendritic cells, neutrophils and other cells in liver.^68^ These cells all participant in the process of liver injury, but whether they can induce pyroptotic cell death to regulate liver inflammation and fibrosis is still unknown and needs further investigation.

## 4 PROMISING ANTIFIBROTIC TREATMENTS AGAINST PYROPTOSIS IN LIVER

Till date, there are no effective treatments for liver fibrosis, it is particularly necessary to explore specific therapeutic targets. According to the identified pathogenesis of liver fibrosis, current studies on the potential therapies mainly focuses on removing exogenous and endogenous harmful factors, inhibiting HSCs activation and transdifferentiation, reducing ECM generation and accelerating its degradation. With the discovery of increasing evidence on the role of pyroptosis in liver fibrosis, targeting pyroptosis may be a prospective therapeutic method. Therefore, it is possible that regulating each centre of pyroptosis negatively can alleviate liver inflammation and fibrosis. Considering that NLRP3 inflammasome is the most important trigger of pyroptosis, inhibiting NLRP3 activation directly or suppressing its downstream signalling pathway (caspase‐1, IL‐1β and IL‐18) are widely regarded as the two main strategies of inhibiting pyroptosis.

NLRP3^−/−^ mice represent less fibrotic lesion in liver under the condition of carbon‐tetrachloride or thioacetamide compared with wild‐type mice.^36^ MCC950, initially called cytokine release inhibitory drugs, is a selective inhibitor of NLRP3 inflammasome. It can interrupt the activation of NLRP3 specifically other than AIM2, NLRP1 and NLRC4 in canonical and noncanonical pyroptotic pathway.^69^ In cholestatic liver injury mice model established by ligating bile duct, the application of MCC950 can decrease the production of proinflammatory cytokines and restrain the infiltration of neutrophils and decrease hepatic cell death obviously. This study suggested that MCC950 attenuated cholestatic liver injury and liver fibrosis via inhibiting NLRP3 activation and assembly.^70^ A similar phenomenon and mechanism can be observed in two models of NLAFD, one of which is C57BL/6 mice fed with methionine/choline deficient diet, and another is foz/foz mice fed with atherogenic diet.^71^ The activation of P2X7 caused by extracellular ATP can also trigger NLRP3 inflammasome. A438079, a specific inhibitor of P2X7, could also suppress liver inflammation and decrease collagen formation and deposition in CCl4‐induced liver fibrosis mice.^72^ Liraglutide, a treatment drug of diabetes, also can inhibit the NLRP3 inflammasome activation and the expression of downstream pathway molecules of pyroptosis in hepatocytes, which has been demonstrated to protect against NASH in human yet.^73^, ^74^ Besides, some traditional Chinese medicines and herbal extracts, such as silybin, dihydroquercetin, baicalein and so on, can also inhibit pyroptosis by interfering with NLRP3 activation and assembly, and ultimately exert anti‐inflammatory and antifibrotic effects.^75^ Despite this, only a few drugs that directly target NLRP3 to attenuate liver fibrosis have been discovered, more researches are needed urgently.

Many caspases are involved in programmed cell death including pyroptosis and apoptosis. The activity of caspases is significantly increased after inflammasome activation in both clinical patients and animal models of liver fibrosis. As inhibitors of caspase VX‐166 and IDN‐6556 can both reduce histopathological changes in MCD‐induced mice and BDL mice respectively, which are mediated by inhibiting hepatocytes apoptosis.^76^, ^77^ Recent study proved that caspase‐11 deficiency can ameliorate liver inflammation and fibrosis by inducing hepatocytes pyroptosis in NALFD mice.^78^ Therefore, blocking hepatocyte pyroptosis with the administration of caspase inhibitors may be a target for pharmacotherapy of liver fibrosis in the future. But it is seem to be unsuccessful to treat human liver injury models with caspase inhibitors due to the long‐term use of caspase inhibitors can drive a more severe, persistent and uncontrollable inflammatory response.^79^ So, to investigate the multiple role of caspase inhibitors on cell survival and death is particularly important before the introduction of caspase inhibitors into the clinic.

Interleukin‐1 is the most widely studied downstream mediator of NLRP3 inflammasome. It can dose‐dependently promote the proliferation and transdifferentiation of HSCs. Inhibiting IL‐1 signalling pathway is perceived as an optimal treatment to suppress NLRP3 activation by so far. In this pathway, IL‐1β, type I IL‐1 receptor (IL‐1R1) and IL‐1 receptor antagonist (IL‐1Ra) are crucial regulatory factors. Recombinant IL‐1 receptor antagonist and humanized monoclonal anti‐IL‐1β antibodies, recognized as an important inhibitor of IL‐1, are used in some inflammatory and autoimmune diseases. Petrasek has discovered that IL‐1β pathway is necessary for the progression of liver inflammation and fibrogenesis in ASH by using IL‐1Ra treated mice as well as caspase‐1/ASC/IL‐1R1 deficiency mice, while the treatment of recombinant IL‐1Ra (anakinra) can ameliorate liver injury by inhibiting IL‐1 signalling.^39^ In addition, anakinra can significantly reduce ECM deposition, inhibit HSC activation, downregulate serum levels of fibrosis markers, and promote regression of liver injury and fibrosis in CCL4 and dimethylnitrosamine induced animal models.^80^, ^81^ However, in Abcb4 deficient mice, as a model of chronic cholestatic liver disease, anakinra do not exhibit antifibrotic effect.^82^ Therefore, the protective roles of interfering IL‐1 in various hepatic fibrosis models and even liver disease patients need deeper confirmation and exploration. Besides, IL‐18 antibody was discovered to restrain HSC proliferation stimulated by HCV serum.^83^

As aforementioned, GSDMD is the executor of pyroptosis, but researches aimed at this molecule in liver fibrosis is not very too much. Xu discovered that with the administration of MCD, the expression level of α‐SMA, TGF‐β1 and hydroxyproline, as well as the degree of steatosis, inflammation and fibrosis are examined lower in GSDMD^−/−^ mice compared with wild‐type mice.^84^ Necrosulfonamide, an anti‐necrosis molecule, binds to GSDMD directly and inhibits GSDMD‐NT pore formation to prevent pyroptosis in a model of sepsis.^85^ It suggests that targeting GSDMD inhibitors is a potential protective measure in treating various liver diseases.

## 5 CONCLUSION

In summary, pyroptosis presents a dual effect in many diseases. It defences against exogenous infections and endogenous danger signals in the early stage of injury, on the contrary, uncontrolled pyroptotic cell death can cause amplified inflammatory responses and followed by pathological changes. Blocking pyroptosis related molecules such as NLPR3, caspases and IL‐1, will impede the development of liver disease and offer a prospective therapeutic target for liver fibrosis. However, due to the immune clearance and defence effects of pyroptosis, improper suppression of pyroptosis may also bring a series of side effects. The mechanism of inflammasome signalling in liver fibrosis still remains unknown; thus, further researches are needed to elucidate the role of different types of inflammasome in various liver injuries and develop effective novel drugs inhibiting pyroptosis for liver fibrosis.

## CONFLICT OF INTEREST

The authors have no conflict of interest to declare.

## AUTHOR CONTRIBUTIONS

**Hui Yang:** Conceptualization (equal); Writing – original draft (lead); Writing – review & editing (equal). **Juan Wang:** Writing – review & editing (equal). **Zhenguo Liu:** Conceptualization (equal); Validation (equal); Writing – review & editing (equal).

## Data Availability

Data sharing is not applicable to this article as no new data were created or analysed in this study.
